# Transplantation of Human Neural Progenitor Cells Expressing IGF-1 Enhances Retinal Ganglion Cell Survival

**DOI:** 10.1371/journal.pone.0125695

**Published:** 2015-04-29

**Authors:** Jie Ma, Chenying Guo, Caiwei Guo, Yu Sun, Tiffany Liao, Ursula Beattie, Francisco J. López, Dong Feng Chen, Kameran Lashkari

**Affiliations:** 1 Schepens Eye Research Institute, Massachusetts Eye and Ear Infirmary, Department of Ophthalmology, Harvard Medical School, Boston, 02114, MA, United States of America; 2 Ophthalmology DPU, RD. Alternative Discovery & Development, GlaxoSmithKline, King of Prussia, PA, 19406, United States of America; Hanson Institute, AUSTRALIA

## Abstract

We have previously characterized human neuronal progenitor cells (hNP) that can adopt a retinal ganglion cell (RGC)-like morphology within the RGC and nerve fiber layers of the retina. In an effort to determine whether hNPs could be used a candidate cells for targeted delivery of neurotrophic factors (NTFs), we evaluated whether hNPs transfected with an vector that expresses IGF-1 in the form of a fusion protein with tdTomato (TD), would increase RGC survival *in vitro* and confer neuroprotective effects in a mouse model of glaucoma. RGCs co-cultured with hNP^IGF-TD^ cells displayed enhanced survival, and increased neurite extension and branching as compared to hNP^TD^ or untransfected hNP cells. Application of various IGF-1 signaling blockers or IGF-1 receptor antagonists abrogated these effects. *In vivo*, using a model of glaucoma we showed that IOP elevation led to reductions in retinal RGC count. In this model, evaluation of retinal flatmounts and optic nerve cross sections indicated that only hNP^IGF-TD^ cells effectively reduced RGC death and showed a trend to improve optic nerve axonal loss. RT-PCR analysis of retina lysates over time showed that the neurotrophic effects of IGF-1 were also attributed to down-regulation of inflammatory and to some extent, angiogenic pathways. This study shows that neuronal progenitor cells that hone into the RGC and nerve fiber layers may be used as vehicles for local production and delivery of a desired NTF. Transplantation of hNP^IGF-TD^ cells improves RGC survival *in vitro* and protects against RGC loss in a rodent model of glaucoma. Our findings have provided experimental evidence and form the basis for applying cell-based strategies for local delivery of NTFs into the retina. Application of cell-based delivery may be extended to other disease conditions beyond glaucoma.

## Introduction

Stem or progenitor cells can be used to restore function in two distinct ways: direct integration into target tissue and/or as carriers of biologically active factors. In the first paradigm, multipotent or unipotent cells differentiate into a specific cell type after reaching the target site after transplantation [1,2,3]. For instance, previous studies have found that rod precursors can successfully integrate into adult or degenerating retina [1,2,4] and form classic triad synaptic connections with second-order bipolar and horizontal cells [2]. In the second paradigm, cells are able to secret NTFs in culture media [5] or in the target location leading to the intended effects in a paracrine manner with mild direct cellular integration [5,6,7]. Studies regarding this paradigm confirm that RGC and axon survival can be increased both *in vitro* and *in vivo* by transplanting human dental pulp stem cells [6] or bone marrow-derived mesenchymal stem cells [5,6,7] by intravitreal injection. In general, grafted cells remain viable for a relatively short period within the target area [7,8].

A similar concept has been applied to retinal neuronal stem/progenitor cells, which can be used for direct replacement of lost cells such as photoreceptors, or to enhance retinal survival after injury through delivery of NTFs. Progenitor-like cells of the retina generally include cells from the ciliary marginal zone and Müller glia [9,10]. We have previous described a retinal neuronal cell line (hNP) whose lineage is strictly restricted to a neuronal and not glial phenotype. Upon differentiation, these cells develop RGC-like characteristics *in vitro* and *in vivo* after induction by retinoic acid [11]. After intravitreal injection, hNPs penetrate and integrate into the host’s inner retina, mostly within the RGC and nerve fiber layers, and extend up to the inner nuclear layer.

We investigated whether hNPs could fulfill one or both paradigms (cell replacement and trophic effects) in a glaucomatous model of RGC injury. To enhance their trophic effects, we stably transfected hNPs with a vector to secrete IGF-1, a known NTF, in the form of a fusion protein with TD. It has been shown that intravitreal injection of IGF-1 inhibits secondary cell death in axotomized RGCs [12]. In addition, *in vitro* [13,14] and *in vivo* [15,16] studies have showed that IGF-1 is developmentally-regulated and its expression in the retina dramatically decreases after birth [17].

Based on these observations, we postulated that IGF-1 would enhance the survival of RGCs and maintain regional density of axons despite the glaucomatous environment. For this purpose, we utilized a model in which elevation of intraocular pressure (IOP) induced by injection of microbeads in the anterior chamber of eyes yields a reproducible loss of RGCs [18,19].

Given that IGF-1 has a very short half-life of about half day [20,21], without a delivery system, it would require multiple intravitreal injections to maintain a therapeutically relevant level that would elicit its trophic effects. To overcome this, we opted for a cell-based system that provided sustained delivery of IGF-1. hNPs were used to locally deliver biologically active IGF-1 in the form of a fusion protein with TD to facilitate its detection *in situ*. The purpose of these experiments was to test the hypothesis that hNPs could be used as a means of local delivery for IGF-1 to the host retina. We evaluated whether hNPs could be stably transfected to express sustained levels of biologically active IGF-1 and explored visualization of the secreted protein and assess whether secretion of IGF-1 could confer global neuroprotection of RGCs both *in vitro* and in experimentally induced stress such as that observed in a model of rodent glaucoma.

In this study, we show that hNPs (hNP^IGF-TD^) that secrete biologically active IGF-1 in the form of a fusion protein with TD (IGF-TD) selectively enhance survival and neurite outgrowth when co-cultured with P0 mouse RGCs, and that this effect can be abrogated with selective inhibitors. Furthermore, using an established and reproducible model of glaucoma, we show that sustained delivery of IGF-TD by hNP^IGF-TD^ cells effectively protect against loss of RGCs. This neurotrophic effect was not observed in untransfected hNPs and hNPs that secrete only TD (hNP^TD^). Analysis of signal pathways by RT-PCR suggests that at least some of the neurotrophic mechanisms of IGF-1 may be related to its anti-inflammatory activity. These findings provide experimental evidence and form the basis for applying cell-based strategies for local delivery of NTFs into the retina.

## Materials and Methods

### Ethics Statement and Animals

This study was approved by the IACUC of the Schepens Eye Research Institute/Mass. Eye and Ear Infirmary for use of animals and by the committee on microbial safety, COMS, at Harvard University. This study adheres to the Helsinki Agreement for clinical studies and use of clinical materials for research. This study was also reviewed and approved by the IRB of Schepens Eye Research Institute /Massachusetts Eye and Ear Infirmary, Harvard Medical School. The study proposal, consent form and method of obtaining consent were approved by the IRB. Each participant was given ample time to read and understand the IRB-approved consenting form prior to his/her surgical procedure. Each subject’s questions and concerns were addressed. A written consent was obtained from each participating subject and each subject received a copy of the signed consent form. We carefully followed the protocol to perform our animal experiments. After the microbead injection and cell transplantation, all animals were closely monitored to ensure no observable signs of inflammatory responses (opaque cornea, corneal edema, iris exudation and synechaie formation) or overt damage in the anterior segment or cataract formation. All efforts were made to minimize animal suffering, to reduce the number of animals used, and to utilize alternatives to in vivo techniques.

### Transfection of hNPs

hNPs were previously isolated from human persistent fetal vasculature retrolental membranes dissected during vitreoretinal surgery from a few young donors. These membranes were cultured according to an established protocol [11]. The coding sequences of IGF-TD or TD were inserted into a pJ603-neo plasmid backbone (DNA2.0, Menlo Park, CA), generating a fusion protein with TD tagged to the C-terminus of IGF-1, or generating TD protein alone, respectively. Gaussia luciferase signal peptide connected at the N-terminus was used to improve IGF-TD or TD expression and secretion [22,23]. Plasmids were transfected into DH5α Competent *E*. *Coli* cells, expanded and purified using the EndoFree Plasmid Maxi Kit (Qiagen, USA). Cells were seeded onto 6-well plates at 1 × 10^5^ cells/well. The next day, the culture medium in each well was replaced with 1 ml the transfection complex (60 μl Lipofectamine 2000, Invitrogen, Carlsbad, CA), 240 μl plasmid (about 650 ng/ml), and serum-free X-vivo medium (Lonza, Watersville, MD, USA). The transfection medium was replaced with regular growth medium comprised of X-vivo medium supplemented with 10% of fetal bovine serum (FBS, Invitrogen), 1:50 B27 (Invitrogen), 1:100 N2 (Invitrogen), 10 ng/ml basic fibroblast growth factor (bFGF; Invitrogen), 20 ng/ml epidermal growth factor (EGF; Invitrogen), and 50 μg/ml nystatin (Sigma, St. Louis, MO, USA) after a 5 hr incubation at 37°C (95% of O_2_, 5% of CO_2_).

### Immunocytochemical Analysis

hNP^IGF-TD^, hNP^TD^, and untransfected hNPs were grown and maintained in X-vivo media supplemented with FGF-2 and EGF [17]. Cells were washed in plain X-vivo media and then seeded on 96-well plates using the same procedure as described above and incubated for 48 hours. Cell were then fixed in 4% paraformaldehyde (20 min), washed in 1× phosphate buffered saline (PBS), blocked with the blocking buffer (Li-Cor, Odyssey, Lincoln, NE) with a supplement of Triton X-100 (2%, Sigma, St. Louis, MO) at room temperature (30 min), incubated in primary antibody solution at 4°C overnight and then rinsed in PBST three times (10 min of each) before being incubated with a secondary antibody solution at room temperature (30 min). Cells were visualized under an inverted fluorescence microscope (Olympus 1X51, Japan). Primary antibodies included goat anti-mouse IGF-1 (1:400, B&D systems, MN) and rabbit anti-red fluorescent protein (for TD; 1:300, Rockland Immunochemicals, Rockland, PA). Secondary antibodies included FITC-bound anti-goat and rabbit (1:500 each, Chemicon).

### Confirmation of IGF-1 Expression in Transfected and Native hNPs

NP^IGF-TD^, hNP^TD^, and untransfected hNPs were seeded onto 6-well plates in serum-free condition, incubated for 48 hours and lysed for total RNA extraction using RNAeasy Plus Mini Kit (Qiagen, Valencia, CA). cDNA was synthesized using the SuperScript III First-Strand Synthesis System (Life Technologies, USA). Quantitative RT-PCR was applied to check mRNA expression of IGF-1. In brief, 0.5 μl cDNA, 1 μl pre-designed primers of IGF-1 or GAPDH, 8.5 μl RNase/DNase-free water and 10 μl KAPA SYBR FAST reaction buffer (Kapa Biosystems, USA) were loaded into 96-well PCR plates in triplicate determinations. A non-template control was included in the experiment to estimate DNA contamination of isolated RNA and reagents.

Western blot analysis was performed to confirm expression of IGF-TD fusion protein. Two days post-transfection, hNP^IGF-TD^, hNP^TD^, and untransfected hNP cells were lysed to extract total protein using 1× RIPA buffer (Cell Signaling) containing 1 mM phenylmethylsulfonyl fluoride, 1× Protease inhibitor cocktail and 1× EDTA (Thermo Scientific, Rockford, IL). Protein lysates were loaded on 4–20% precise pre-casted PAGE gels (Thermo Scientific, Pittsburgh, PA) and subjected to electrophoresis. Gels were transferred to nitrocellulose membranes using a semidry blotter (Bio-Rad, Hercules, CA) for immunoblot analysis. Membranes were blocked with blocking buffer for 1 hr at room temperature, incubated with primary antibodies (overnight, 4°C) including goat anti-mouse IGF-1 (B&D systems, MN, 1:400) and rabbit anti-GAPDH (1:400, Rockland Immunochiemicals) diluted with the blocking buffer and PBST (volume ratio 1:1). Membranes were washed twice in PBST (10 min of each), incubated with secondary antibodies (1:3,000, anti-goat IRDye 800CW, Odyssey) for 1 hr, and washed twice in PBST (10 min of each). Fluorescent protein bands were visualized on the Odyssey Infrared Imaging System (Odyssey).

### ELISA Analysis of Secreted IGF-TD and TD in Transfected hNPs

Secreted IGF-1 (as component of the IGF-TD protein) and TD were measured in hNP^IGF-TD^, hNP^TD^ and hNP cells using a modified ELISA procedure [24,25]. Briefly, a 96-well Elispot plate was coated by sodium carbonate buffer (50 μl/well) and incubated overnight at 4°C. Conditioned media collected from cells on days 0, 1, 3, 5 and 7 were placed in wells of the Epispot plates and incubated overnight to capture the respective proteins. Plates were then briefly rinsed and incubated with blocking buffer (200 μl/well, 10% FBS in 1× PBS) at room temperature for 2 hr. Wells were washed twice with 1× PBS and incubated with goat anti-mouse IGF-1 antibody (1:400) at 4°C overnight. Wells were then washed with 1× PBST three times (5 min each time) and incubated with chick anti-goat HRP-conjugated secondary antibody (Sigma, 1:5000). After washing twice with 1× PBST and 1× PBS, TMB (3, 3^’^, 5’ 5^’^-tetramethylbenzidine) was added to the wells and incubated in the dark for 15–20 min. The reaction was stopped with H_2_SO_4_ (2 M) and the plate was quickly read at OD 450 nm using a microarray reader system (GENios XFLUOR4, Tecan, Männedorf, Switzerland). Recombinant mouse IGF-1 protein (Sigma; 10–250 ng/ml) solutions dissolved in the same diluent were used for as reference standards.

### RGC Survival and Neurite Outgrowth Assays

P0 C57BL/6 mouse pups were euthanized with CO_2_. Eyes were enucleated and retinas were dissected and placed in cold (4°C) Hank’s buffer (Life Technologies) containing 1× Penicillin-Streptomycin-Glutamine (Life Technologies), and digested in 20 U/ml papain solution containing 100 U/ml DNase I at 37°C for 15 min. The reaction was stopped with 5 mg/ml ovomucoid protease inhibitor containing 5 mg/ml albumin. Lysates were triturated several times and centrifuged (252 RCF of 10 cm rotor for 5 min at 4°C). Cell pellets were washed once and maintained in 800 μl washing buffer (0.5% BSA, 2 mM EDTA in 1× PBS). Thy1.2 (CD90.2) microbeads and MACS magnetic separation system (Miltenyi Biotec, Cambridge, MA) were used to isolate RGCs following the manufacturer’s instructions. RGCs in the filtrate were centrifuged (252 RCF of 10 cm rotor for 5 min at 4°C) and re-suspended in culture medium (Neurobasal-A medium supplemented with 25 μM L-glutamic acid, 1 mM L-glutamine, 100 U/ml penicillin, 100 μg/ml streptomycin, 1× B-27, 5 μg/ml insulin, 50 ng/ml BDNF, 50 ng/ml CNTF and 1 μM forskolin; Life Technologies). ß-III tubulin (Millipore, 1:800) was applied to check the purification of RGCs.

hNP^IGF-TD^, hNP^TD^, and untransfected hNPs were seeded onto cell culture inserts (0.4 μm pore size, BD Falcon) and incubated. On day 3, RGCs were spread onto 12-well plates pre-coated with Poly-D-Lysine (Millipore, 0.1 mg/ml) and merosin (Millipore, 5 μg/ml), and 200 μl of RGC culture medium was added into every well. Culture media in the inserts were replaced with 200 μl RGC culture medium before being transferred to the wells. The plates were maintained in an incubator (37°C, 95% of O_2_ and 5% of CO_2_). In some experiments, the following reagents were added into the culture medium immediately after RGCs were seeded: IGF-1 receptor antagonist (H-1356, Bachem, 40 μg/ml), a competitive inhibitor of the IGF-1 receptor [14,26,27,28], IGFBP (IGF-binding protein) inhibitor, NBI-31772 (Millipore, 10 μM), which disrupts the binding of IGF-1 with all six IGFBPs [13], and a blocking antibody to IGF-1 receptor (IGF-1R, 1:250, R&D Systems); these agents were used to explore IGF-1 signaling.

Co-culture inserts and culture media were removed on day 3. RGCs were washed with 1× PBS and stained with CalceinAM and EthD-1 (LIVE/DEAD Viability/Cytotoxicity kit, Life Technologies) for 40 min at room temperature. Images of 4–6 40x fields were randomly selected throughout each well and examined under an Olympus inverted fluorescence microscope. Digitized images were counted using ImageJ 1.46 (National Institutes of Health, Bethesda, MD) and survival rates were calculated as [live cells / (live + dead cells)] × 100%. RGCs in some other wells were fixed with 4% paraformaldehyde for 15 min and then incubated with rabbit anti-mouse ß-III tubulin (1:800) overnight (4°C) and subsequently incubated with secondary goat anti-rabbit Cy3 (1:800) for 1 hr. Neurite lengths were measured using ImageJ 1.46.

### Induction of Microbead Model of Murine Glaucoma and Intravitreal Transplantation of hNPs

C57BL/6 mice (4–6 weeks old) were divided into five groups, of which 4 groups received injections of microbeads and one group received injection of saline into the anterior chamber of one eye (Table 1). This procedure was modified from previous studies [29,30]. In brief, mice were anesthetized by intraperitoneal injection of a ketamine/xylazine (120 mg/kg /12 mg/kg) mixture (Phoenix Pharmaceutical, Inc., St. Joseph, MO). Anesthesia was supplemented by topical proparacaine HCl (0.5%; Bausch & Lomb, Tampa, FL). A small volume of microbeads (Life Technologies; 2 μl of 6.0 × 10^6^ beads/ml) with mean diameter of 15 μM was injected into the anterior chamber of four groups of mice using a glass micropipette connected to a Hamilton syringe. Saline was injected into the anterior chamber of the fifth group of mice using similar technique. Baseline IOPs were checked 2 days prior to the microbead injection, and every other day thereafter. Each measurement was consistently made in the mornings in both eyes of each mouse using a tonometer (TonoLab, Colonial Medical Supply, Espoo, Finland) under topical anesthesia. Every reading on the tonometer was averaged from six measurements by an internal program. The mean of six readings from each eye was used to calculate the IOP.

**Table 1:** Mouse treatment groups receiving different injections.

Groups	Anterior chamber	Vitreous cavity
1	Saline	Saline
2	Microbeads	Saline
3	Microbeads	hNP
4	Microbeads	hNP^TD^
5	Microbeads	hNP^IGF-TD^

hNP: human neuronal progenitor cells; hNP^TD^_:_ cells expressing TD; hNP^IGF-TD^: cells expressing IGF1-TD fusion prote

Intravitreal injection of cells was performed one day after microbead injection. hNP^IGF-TD^, hNP^TD^, and untransfected hNPs were incubated overnight in plain X-vivo medium. One hour before injection, cells were collected from flasks by gentle trituration, washed twice in 10 ml of same medium and centrifuged. The cell pellets were resuspended and diluted to a final concentration of 1 × 10^5^ cells/μl in Hank’s Balanced Salt Solution and kept on ice until transplantation. Trypan blue dye exclusion assay was performed on cell suspensions prior to the transplantation to ensure viability and showed greater than 90% cell survival. In order to study rescue effects on RGCs, the aforementioned cell groups or saline were intravitreally injected into the four groups of mice that had already received microbead injections (Table 1). During this procedure, mice were deeply anesthetized and pupils were dilated with 0.5% proparacaine and 0.5% topical tropicamide solution (both, Bausch & Laumb, Rochester, NY). Cell suspensions (2 μl) were injected into the vitreous cavity of the right eye using a glass micropipette connected to a Hamilton syringe under direct observation through the operating microscope [11].

### Quantification of RGC Loss and Detection of Transplanted hNPs

RGC loss was evaluated by examining and imaging retinal flatmounts collected from all groups. Mice were observed for one month after which they were sacrificed and eyes were enucleated. Retinas were dissected from eye cups under a dissecting microscope and fixed in 4% paraformaldehyde for 2 hrs. Retina flatmounts were washed in 1× PBS for 30 min, incubated in blocking buffer at room temperature for 1 hr, and incubated with primary rabbit anti-mouse ß-III tubulin (Millipore, 1:400) or with primary anti-mouse Brn3a (Millipore, MAB1585, 1:20) at 4°C, overnight. Retina flatmounts were then washed with 1× PBST three times (10 min each time), incubated with secondary goat anti-rabbit Cy3 (1:800; Chemicon) or secondary goat anti-mouse FITC antibody (1:800; Chemicon) for 1 hr, covered with mounting medium (Vectashield; Vector Lab, Burlingame, CA) and imaged under the Leica TSC SP5 confocal microscope. To assess RGC counts, retina flat mounts were divided into 4 quadrants: superior, temporal, nasal, and inferior. RGC density was calculated based on expression of ß-III tubulin, representing the number of host RGCs per quadrant. In parallel, cryosections were prepared from eye cups from each group and immunostained with ß-III tubulin to evaluate the RGC and nerve fiber layers in cross section. Alternatively, cryosections were immunostained with Brn3a antibody to detect RGC nuclei and confirm ß-III tubulin staining. Transplanted hNPs were detected on retinal flatmounts by their respective expression of IGF-1, TD and human HLA Class I antigen (Sigma, St. Louis, MO) in retinal flatmounts.

### Quantification of Axons in Optic Nerves Cross Sections

Mouse optic nerve samples from each study group were harvested and fixed with ½ Karnovsky’s fixative (2% of paraformaldehyde and 2.5% of glutaraldehyde in 0.1 M sodium cacodylate buffer). After fixation, samples were rinsed with 0.1 M sodium cacodylate buffer, post-fixed with 2% osmium tetroxide in 0.1 M sodium cacodylate buffer, *en bloc* stained with 2% of aqueous uranyl acetate, then dehydrated with graded ethyl alcohol solutions, transitioned in propylene oxide and embedded in Embed-812 epoxy resin (Tousimis, Rockville, MD) utilizing an automated EMS Lynx 1 EM tissue processor (Electron Microscopy Sciences, Hatfield, PA) and polymerized in silicone molds using an oven set at 60°C. Semi-thin 1 μm cross-sections were cut with Histo diamond knives (Diatome, Hatfield, PA) on a Leica UC7 Ultramicrotome (Leica Microsystems, Buffalo Grove, IL). The myelinated axons within the semi-thin sections were stained with a freshly prepared 0.2 μm filtered aqueous 2% of *p*-paraphenylenediamine solution (Fisher Scientific, Fair Lawn, NJ) for 2 min on a warm plate set at 60°C, then rinsed in tap water for 1 min, and washed 4 times (1 min of each) in deionized water. Stained slides were air-dried then briefly dipped in xylenes and applied with a few drops of mount media and a glass coverslip to cover the sections. Images were collected for a given nerve section at 100× magnification using a Nikon microscope (Eclipse E800, Nikon, Japan) with a DPController software (Olympus, Japan). Axons were counted from each image using ImageJ 1.46. Axonal density was calculated by dividing the number of axons in an individual image by the area. The percentage of axon loss was calculated by dividing the mean of axons in each nerve cross section in microbead-injected eyes (Table 1, group 2–5) with the mean of axons in saline-injected (Table 1, group 1) eyes.

### Quantification of the Gene Expression

Previous studies have indicated that IGF-1 can lead to retinal vascularization [31,32]. In addition, one of the typical symptoms of glaucoma is the RGC loss and thinning of the nerve fiber layer consistent with a neurodegenerative process. Neurodegenerative process are generally accompanied by activation of apoptotic pathways, which can in turn induce an inflammatory response [33]. Using RT-PCR, we tested whether glaucomatous optic nerve loss was associated with any changes in gene expression of apoptotic, angiogenic and inflammatory pathways. Genes and primers are listed in Table 2. This analysis was performed for all 5 groups. The procedure of qRT-PCR has been addressed in a previous section.

**Table 2:** The list of genes and primers.

Genes	Primers (5’– 3’)
mouse VEGF-A	Forward: TGGTTCTTCACTCCCTCAAATC
	Reverse: CTCTCCTCTTCCTTCTCTTCCT
mouse VEGF-C	Forward: TCTCTGCCAGCAACATTACC
	Reverse: GACACAGCGGCATACTTCTT
mouse VEGF-D	Forward: CACCTCCTACATCTCCAAACAG
	Reverse: CCGCATAAGAAAGAAGCACAATAA
mouse VEGFR-2	Forward: GGACAGCGAGATAGGCTTTAC
	Reverse: TCCACGTGTCTCCATTCTTTAC
Mouse VEGFR-3	Forward: CATCCACCACAGCTCTACATATC
	Reverse: GTGGCTCTGGTCTAACTCTTTC
mouse CD11b	Forward: GTGGTGATGTTCAGGGAGAAT
	Reverse: GGGTCTAAAGCCAGGTCATAAG
mouse TNF-α	Forward: GGGAGAACAGAAACTCCAGAAC
	Reverse: GTTGGACCCTGAGCCATAATC
mouse IL-1ß	Forward: TCATTGTGGCTGTGGAGAAG
	Reverse: GCTTGTGAGGTGCTGATGTA
mouse IP-10	Forward: TCCTAATTGCCCTTGGTCTTC
	Reverse: GCACCTCCACATAGCTTACA
mouse MCP-1	Forward: CTCACCTGCTGCTACTCATTC
	Reverse: TGGATCCACACCTTGCATTTA
mouse MFG-E8	Forward: GGGACATCTTCACCGAATACA
	Reverse: TGCTTCCTTGCTCCTCATATAC
Mouse IGF-1	Forward: AGATGCACTGCAGTTTGTGTGTGG
	Reverse: TCTACAATTCCAGTCTGTGGCGCT
mouse GAPDH	Forward: GTGGCAAAGTGGAGATTGTTG
	Reverse: GTGGTCCAGGGTTTCTTACTC
human IGF-1	Forward: ATGCACTGCAGTTTGTGTGTGGTC
	Reverse: TACAATTCCAGTCTGTGGCGCTCT
human GAPDH	Forward: GGCCTCCAAGGAGTAAGACC
	Reverse: AGGGGTCTACATGGCAACTG

### Statistical Analysis

Statistical analysis was performed using the SigmaPlot (12.5, San Jose, CA). Results are expressed as mean ± SE (standard error of mean) unless otherwise stated in the figure legend. Some of the data were analyzed using either the Mann-Whitney U test or the Kruskal-Wallis test followed by the Dunn’s test for multiple pairwise comparisons between the five groups because the data failed to normality test (Shapiro-Wilk test, *P* < 0.05). In addition, a two-way linear model for treatments and time points was used in a time course study *in vitro*. Data that had a repeated measures component were analyzed by fitting a mixed effects model to the data with either single or double fixed effects (treatment or treatment and time) with a random intercept for the subject. All analyses were conducted after applying a Box-Cox-transformation to the data. Specific transformations used for each data set are described in the legends to the figures. After a significant difference was detected in any of the models, multiple comparisons were conducted by the Tukey’s test on the transformed scale. The analysis was conducted using R studio and R version 3.1.2. *P* values < 0.05 were defined as significant.

## Results

### Generation of hNP Cells exressing IGF-TD and TD

**Fig 1:**
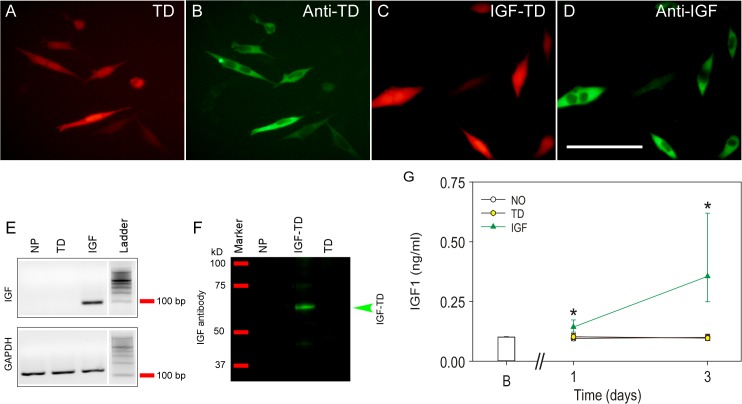
Detection of tdTomato (TD) and IGF1-TD fusion protein in human neuronal progenitor cells (hNP). (A) Expression of TD protein in transfected hNPs by their red fluorescence. (B) TD protein can also be detected by using immunohistochemistry (FITC, green). (C) Expression of IGF-TD fusion protein in hNPs by their red fluorescence. (D) Detection the IGF-1 moiety of the IGF-TD fusion protein in transfected cells by immunostaining (FITC, green). (E) Expression of IGF-1 component of the IGF-TD mRNA in hNP^IGF-TD^ cells by qRT-PCR; IGF-1 mRNA is undetectable in untransfected hNP and hNP^TD^ cells. (F) Western blot analysis of IGF-1 component of the IGF-TD protein in hNP^IGF-TD^ cell lysates confirms the expected molecular weight of approximately 60 kD. IGF-1 was not detected in untransfected hNP and hNP^TD^ cells. (G) IGF-1 levels are higher in the medium of cells transfected with the IGF-TD fusion protein (red triangles). Data are presented as mean ± SE for the day 0 wells (B; blank wells). Symbols correspond to the fitted means ± the 95% confidence limits of the measurements conducted in conditioned medium from the difference hNPs (open symbols; empty vector, yellow symbols; TD-transfected cells, green symbols; IGF-TD—transfected cells). Analysis was conducted by fitting a two way linear model for treatments and time points on the inverse of the data. Multiple comparisons were conducted on the transformed scale by the Tukey test. Asterisks indicate statistically significant differences *versus* all groups at the same time point. Abbreviations: hNP, neuronal progenitor cells; TD, tdTomato; IGF-TD, IGF-1-tdTomato. Scale bar in A—D: 50 μm.

hNP^IGF-TD^ cells were generated by transfecting them with a vector containing the coding sequences of IGF-TD, in which the TD sequence was tagged to the C-terminus of IGF-1 producing a fusion protein. Similarly, hNP^TD^ cells were generated by inserting the TD coding sequence alone. Immunostaining of transfected cells with antibodies against TD or IGF-1 confirmed the expression of TD and IGF-TD proteins in specific cells (Fig 1A–1D). High levels of mouse IGF-1 mRNA was also detected by real time RT-PCR in hNP^IGF-TD^ cells but not in hNP^TD^ or untransfected hNP cells (Fig 1E). The synthesis and secretion of IGF-TD protein by hNP^IGF-TD^ cells were detected from the cell lysates using Western blot analysis (Fig 1F). ELISA was also used to evaluate the concentration of IGF-TD in the conditioned media collected from cultured hNP^IGF-TD^ and hNP^TD^ cells at 1 and 3 days post-transfection. The concentration of IGF-TD protein gradually increased to about 0.5 ng/ml on day 3 (Fig 1G). In contrast, there were very low levels of IGF-1 in hNP^TD^ or hNPs (Fig 1G).

### hNP^IGF-TD^ Cell Cocultures Enhance Survival and Neurite Outgrowth of primary RGCs

**Fig 2:**
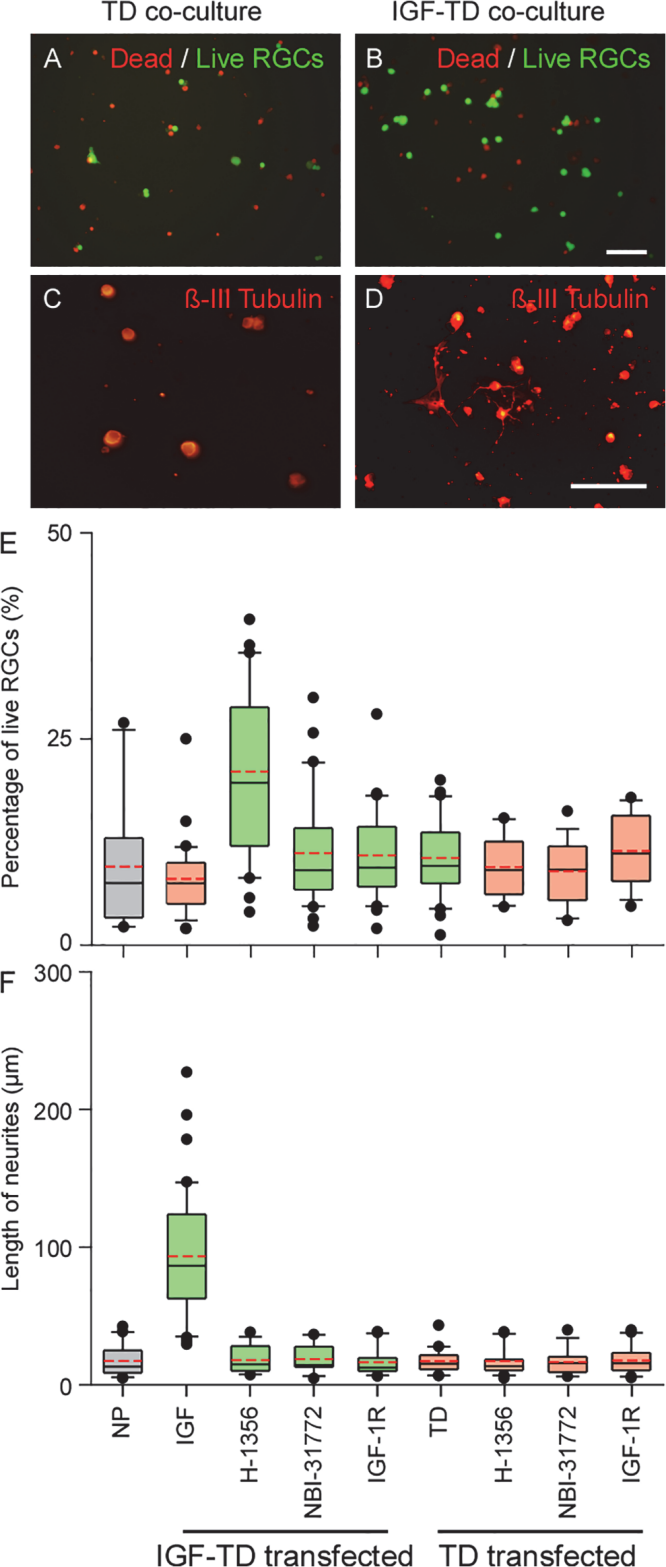
The survival rate and neurite outgrowth of primary RGCs co-cultured with transfected hNPs under various conditions. Dead/live (green/red fluorescence) cell analysis of primary RGCs co-cultured with hNP^TD^ (A) and hNP^IGF-TD^ (B) cells shows increased live cells in the latter group. ß-III tubulin staining of co-cultured cells indicates that neurites were rarely observed in RGCs co-cultured with hNP^TD^ cells (C) as compared with RGCs co-cultured with hNP^IGF-TD^ cells (D) (Cy3, orange-red fluorescence). (E—F) Quantification of survival rate and neurite length in RGCs co-cultured with hNP^IGF-TD^ or hNP^TD^ cells in presence and absence of IGF antagonists Data was analyzed with Mann-Whitney U test. RGC survival rate was significantly higher in hNP^IGF-TD^ co-cultures; IGF antagonists reduced both survival rate and neurite outgrowth of RGCs. The boxes in (E) and (F) represent the 0.25, median and 0.75 quantiles. On either side of the box, the whiskers extend to the minimum and maximum. Detailed index of each transfection is presented in the text. The red dash line in each box is the mean value. Abbreviations: NP, neuronal progenitor cells; TD, neuronal progenitor cells expressing TD (hNP^TD^); IGF-1, neuronal progenitor cells expressing IGF-TD fusion protein (hNP^IGF-TD^); Scale bar: 200 μm (A—B) and 50 μm (C—D).

A co-culture system was used to evaluate the effects of secreted IGF-TD on survival and neurite outgrowth of RGCs. In presence of hNP^IGF-TD^ cells, survival of RGCs (22 ± 2% cells/field) was significantly higher than RGCs co-cultured with hNPs^TD^ (11 ± 3% cells/field) and untransfected hNPs (10 ± 1% cells/field; *P* < 0.05; Fig 2A and 2B and Fig 2E). RGCs co-cultured with hNP or hNP^TD^ exhibited similar survival rates (Fig 2E). When co-cultured with hNP^IGF-TD^ cells, RGCs extended long neurites with an average length of 93 ± 7 μm (*P* < 0.05, Fig 2F), while RGCs co-cultured with hNP^TD^ or hNP cells, developed average neurite lengths of 17 ± 2 μm and 17 ± 3 μm, respectively (*P* > 0.05, Fig 2C and 2D and Fig 2F). Moreover, RGCs co-cultured with hNP^IGF-TD^ cells also produced more neurites per cell body, averaging 2 ± 0 neurites/cell, while those co-cultured with hNP^TD^ or hNP cells displayed an average of 1 neurite/cell each (*P* < 0.05). These observations confirm that IGF-TD secreted by hNP^IGF-TD^ cells was able to enhance the survival and neurite outgrowth of cultured primary RGCs. Analysis was conducted by fitting a two way linear model for treatments and time points on the inverse of the data. Multiple comparisons were conducted on the transformed scale by the Tukey test. Asterisks indicate statistically significant differences *versus* all groups at the same time point.

### IGF-1 Inhibitors Abrogate RGC Survival and Neurite Outgrowth

To confirm the direct effects of IGF-TD on RGC survival and neurite outgrowth in hNP^IGF-TD^ co-cultures, we applied various antagonists: H-1356 is an IGF-1 analog that competitively binds and blocks IGF-1R signaling; NBI-31772, a tyrosine kinase receptor disrupts the binding of IGF-1 to IGF-1 binding proteins (IGFPBs); and a neutralizing antibody to IGF-1R. Our observations indicate that application of H-1356 completely eliminated the effects of hNP^IGF-TD^ on RGC survival and neurite outgrowth. Similarly, NBI-31772 and a neutralizing antibody to IGF-1R also independently and completely blocked these effects. The rates of cell survival and average neurite lengths of RGCs co-cultured with hNP^IGF-TD^ in presence of these inhibitors were not significantly different from the observed rates for RGCs co-cultured with hNP^TD^ or hNP cells (Fig 2E and 2F).

### Rescue of RGCs via IGF-1 Signaling in the Retina

**Fig 3:**
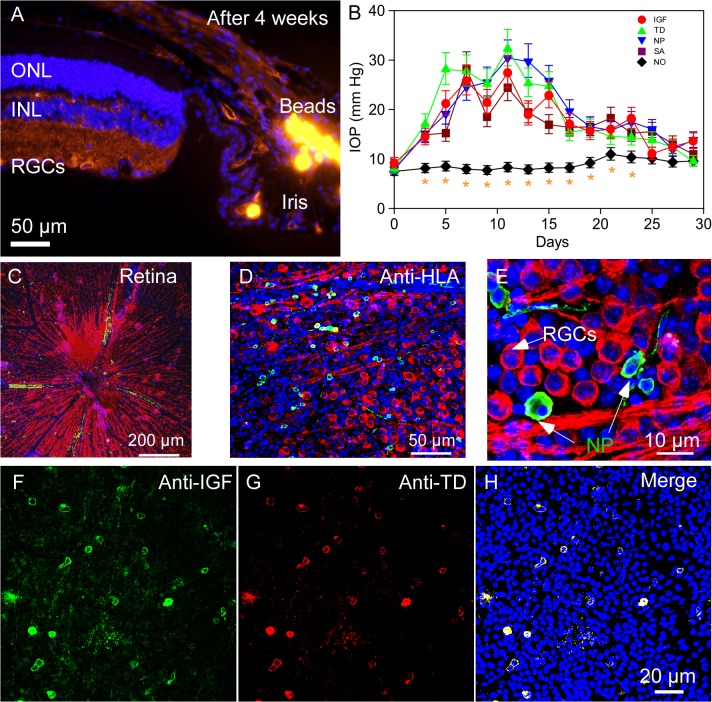
Microbead injection into the anterior chamber of C57BL/6 mice to induce elevation of IOP and glaucoma model. (A) After 4 weeks, microbeads can be detected in the Schlemm’s canal (orange fluorescence on the right side of the photograph). (B) Fitted geometric mean IOP changes with the 95% confidence limits in all microbead injected and control groups. (group 1–5). Saline-injected group showed mean IOPs of ≤ 10 mmHg (group 1). Microbead injected eyes (group 2–5) showed rapid and steady rise in IOPs peaking between 5–12 days and sustained elevated IOPs during the one-month study period. Data were analyzed by fitting a mixed effects model to the logarithm of the data with two fixed effects (treatment and time) and the subject as a random intercept. Multiple comparisons were conducted using the Tukey test on the fitted data. Asterisks denote statistically significant differences *versus* all groups within the same time point. (C) Representative retinal wholemount after intravitreal hNP transplantation imaged on confocal microscopy. Red fluorescent structures (ß-III tubulin) represent host RGCs and their nerve fibers. (D) Green fluorescent dots (FITC, HLA Class I antigen expression) present hNPs that have penetrated the host retina. (E) High-resolution images of retinal flatmounts. (F, G) hNPs expressing IGF-TD can also be detected by their expression of IGF-1 (FITC-green) and TD (Rhodamine-red) components of the fusion protein. (H) Merged image of F and G. Abbreviations: ONL, outer nuclear layer; INL, inner nuclear layer; RGCs, retinal ganglion cells. IGF-TD, transplanted hNP^IGF-TD^ cells after microbead injection; TD, transplanted hNP^TD^ cells after microbead injection. hNP, untransfected hNPs after microbead injection; SA, intravitreal saline (no cells) injection after microbead injection; NO, intravitreal saline injection and saline injection into the anterior chamber (no microbead and cell injection).

We used an established model of murine glaucoma in which injections of microbeads into the anterior chamber of the mouse eye causes blockage of aqueous humor outflow and reproducible elevation of IOP (Fig 3A). Prior to microbead injection, baseline IOPs were measured and averaged 8.17 ± 0.88 mmHg (n = 30). Mice that received control saline injections into their anterior chambers (group 1) exhibited a steady IOP level of 8.64 ± 1.04 mmHg (n = 6) throughout the study period. No significant differences were found among IOPs of saline-injected mice (group 1) and baseline IOP values of other groups (group 2–5, n = 6/group, *P* > 0.05). Significant elevation of IOPs in the microbead-injected mice was observed within 4 days after the injection (between 8–20 mmHg), reaching peak levels around day 10 (ranging between 23.78–32.42 mmHg, Fig 3B). Elevated IOPs gradually dropped off around 2 weeks after injection and returned to the baseline by week 4 (Fig 3B). There were no significant differences in IOP levels among the four microbead-injected groups (group 2–5, all *P* > 0.05). Expectedly, these groups exhibited higher IOPs compared with the saline-injected group (all *P* < 0.05, Fig 3B). Our observations showed that injection of microbeads into anterior chamber effectively induced reproducibly transient elevation of IOP.

Mouse group 2–5 (n = 6/group, Table 1) received intravitreal injections of one of the following components, in the following order, saline, hNPs, hNP^TD^ and hNP^IGF-TD^. Mice were sacrificed on day 30 and retinal flatmounts were prepared and subjected to immunostaining (Fig 3C–3H). Transfected cells were confirmed to be of hNP origin (hNP, hNP^TD^ or hNP^IGF-TD^) using an antibody to human HLA Class I antigen (Fig 3D). Interestingly, some transplanted hNPs displayed neurite-like structures (Fig 3E). Immunostaining with anti-mouse IGF-1 and anti-TD antibody confirmed the expression of IGF-TD and TD by hNP^IGF-TD^ and hNP^TD^ cells, respectively (Fig 3F–3H). Data were analyzed by fitting a mixed effects model to the logarithm of the data with two fixed effects (treatment and time) and the subject as a random intercept. Multiple comparisons were conducted using the Tukey test on the fitted data. Asterisks denote statistically significant differences *versus* all groups within the same time point.

**Fig 4:**
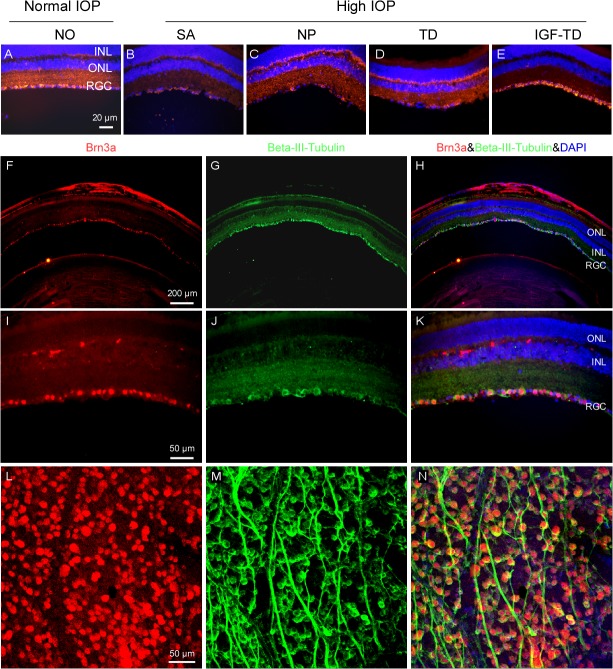
RGC loss in the experimental glaucoma model. (A—E) Retina sections immmunostained for ß-III tubulin (red fluorescence) confirm microbead-induced glaucomatous RGC loss (SA, NP and TD) and RGC rescue in eyes transplanted with hNP^IGF-TD^ cells (IGF-TD). Brn3a antibody (red fluorescence) was used to verify the accuracy of RGC density stained with ß-III tubulin (green fluorescence) on retinal cross sections. (F—K) and retinal flatmounts using confocal microscopy (L—N). RGC population was slightly overestimated (1.7%) using ß-III tubulin staining compared to Brn3a staining, but the difference was not significant (Mann-Whitney U test, *P* > 0.05). Abbreviations: ONL, outer nuclear layer; INL, inner nuclear layer; RGC, retinal ganglion cell; IGF-TD, transplanted hNP^IGF-TD^ cells after microbead injection; TD, transplanted hNP^TD^ cells after microbead injection. hNP, untransfected hNPs after microbead injection; SA, intravitreal saline (no cells) injection after microbead injection; NO, intravitreal saline injection and saline injection into the anterior chamber (no microbead and cell injection). High IOP, elevated intraocular pressure by microbead injection. Scale bar: 50 μm in A—E.

Examination of retinal cross sections from these groups (Table 1) confirmed the expected and comparable loss of host RGCs and thinning of the nerve fiber layer in group 2–4 (Fig 4B–4D) compared to saline-injected control group (group 1, Fig 4A). These included eyes that had been transplanted with hNP^TD^ or hNP cells suggesting that hNPs either do not express adequate intrinsic factors to exert any significant neurotophic effects on RGCs (in this model) or that any amount of human NTFs released by hNPs do not exert adequate neurotrophic effects in the mouse retina. In contrast, mice receiving hNP^IGF^ (Fig 4E) exhibited higher RGC density, which was comparable to saline-injected eyes (Fig 4A).

In order to confirm that ß-III tubulin adequately identified RGCs, Brn3a antibody was used to verify the accuracy of RGC density stained with ß-III tubulin on retinal cross sections (F—K) and retinal flatmounts (L—N). There was good anatomic correlation between the expression of ß-III tubulin and Brn3a in both preparations. RGC population was slightly overestimated (1.7% ± 0.3) with ß-III tubulin staining as with Brn3a staining, but this difference was not significant (Mann-Whitney U test, *P* > 0.05).

**Fig 5:**
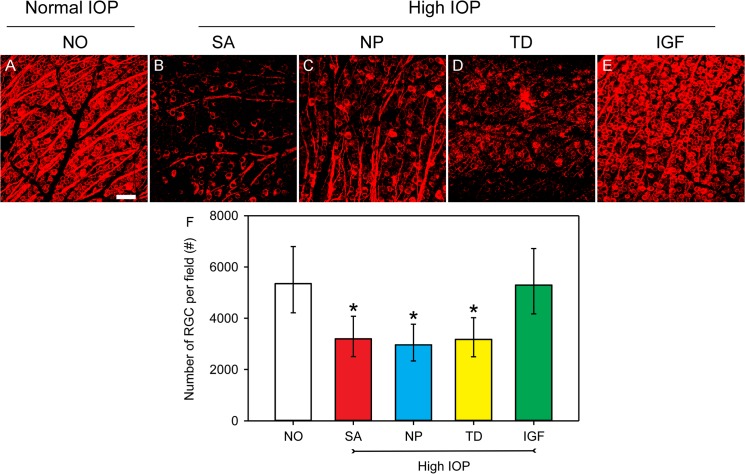
Quantification of RGC density using ß-III tubulin (red fluorescence) in retinal flatmounts under different experimental conditions. (A—E) Confocal images show significant and robust RGC loss is visualized in the saline, hNPs and hNP^TD^ transplanted groups after microbead injections. RGC density and distribution in microbead-injected eyes transplanted with hNP^IGF-TD^ cells (E) were similar to the control group (A). (F) Quantification of RGC density in various experimental groups. Data are presented as the fitted geometric means and the 95% confidence limits. Analysis was conducted using a mixed effects model with a random intercept for each subject followed by the Tukey test on the logarithm of the data. *P* < 0.05 compared to NO group; #, *P* < 0.05 compared to IGF-TD group. Scale bar: 50 μm in A—E. Abbreviations: IGF-TD, transplanted hNP^IGF-TD^ cells after microbead injection; TD, transplanted hNP^TD^ cells after microbead injection. hNP, transplanted untransfected hNPs after microbead injection; SA, intravitreal saline (no cells) injection after microbead injection; NO, intravitreal saline injection and saline injection into the anterior chamber (no microbead and cell injection). High IOP, elevated intraocular pressure by microbead injection.

To quantitatively assess the neuroprotective effect of cell transplants, RGCs were counted in retinal flatmounts after immunostaining with ß-III tubulin. Analysis was conducted using a mixed effects model with a random intercept for each subject followed by the Tukey test on the logarithm of the data. Data are presented as the fitted geometric means and the 95% confidence limits. Panel A in Fig 5 shows RGC distribution in retinal flatmounts of saline into both anterior chambers and vitreous cavity (Group 1, no glaucoma). In this control group, the RGC density estimated at 5352 (95% confidence limits [CL] of 4214 to 6797) RGCs/mm^2^. In mice that received microbead injections to their anterior chambers and saline in their vitreous (glaucoma group, SA in Fig 5F), retinal flatmounts showed an approximate 60% reduction in RGC count, with an estimated density of 3196 (95% CL: 2505–4077) RGCs/mm^2^. This ~60% loss of RGCs corresponds to the expected response to elevated IOP (Fig 5B and SA in panel F). Microbead-injected mice that received untransfected hNPs or hNP^TD^ also exhibited ~60% reductions in RGC density (Fig 5F). The estimated densities for these groups were 2965 (95% CL: 2335–3764) and 3173 (95% CL: 2500–4028) RGCs/mm^2^, respectively (Fig 5C and 5D, and hNP^TD^ in panel F). Microbead-injected mice received hNP^IGF-TD^ exhibited RGC densities of 5293 with 95% CL of 4168 to 6722 RGCs/mm^2^ (Fig 5E and IGF in Fig 5F), the latter being not statistically different from saline-injected control group (SA in Fig 5F; *P* > 0.05). RGC density measurements among microbead-injected eyes for saline, hNPs or hNP^TD^ injected groups were quite similar (all *P* > 0.05) but statistically different from saline and hNP^IGF-TD^ injected groups (Fig 5F). These data indicate that IGF completely restores IOP-induced RGC loss, as IGF-treated animals had ~99% of RGC density observed in the saline injected controls.

**Fig 6:**
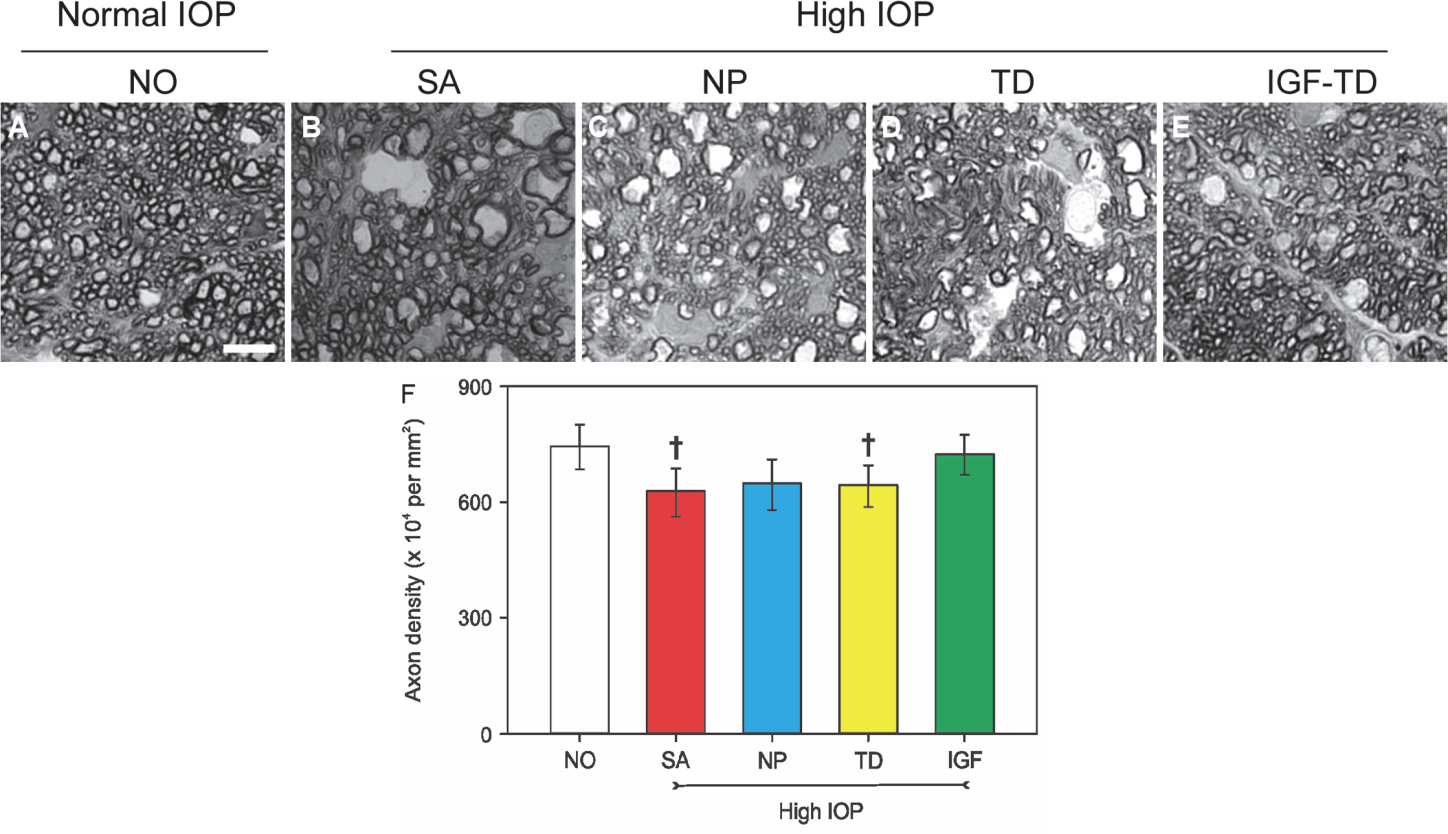
Quantification of axons on semi-thin cross sections of optic nerve under different experimental conditions. (A) Cross section of axons from the intraorbital section of the optic nerve after saline injection (no glaucoma). Significant axon degeneration is observed in the saline, hNPs and hNP^TD^ transplanted groups after microbead injection (B—D). There is better preservation of axons in the hNP^IGF-TD^ transplanted group (E). (F) Quantification of axon cross-sections indicates that transplantation of hNP^IGF-TD^ cells show a trend to enhanced survival of axons in the glaucomatous environment (data are presented as the fitted means and the 95% confidence limits. Analysis was conducted using a mixed effects model with a random intercept for each subject followed by the Tukey test on the square of the data. Crosses indicate a trend (*P* < 0.1) compared to the NO and IGF groups). Scale bar: 5 μm in A—E. Abbreviations: IGF-TD, transplanted hNP^IGF-TD^ cells after microbead injection; TD, transplanted hNP^TD^ cells after microbead injection. hNP, transplanted (untransfected) hNPs after microbead injection; SA, intravitreal saline (no cells) injection after microbead injection; NO, intravitreal saline injection and saline injection into the anterior chamber (no microbead and cell injection). High IOP, elevated intraocular pressure by microbead injection.

RGC axon loss was also quantified by quantifying axonal cross-sections. Data are presented as the fitted means and the 95% confidence limits. Analysis was conducted using a mixed effects model with a random intercept for each subject followed by the Tukey test on the square of the data. In the saline-injected (both anterior chamber and vitreous) group, the axon density was 745 × 10^4^ axons/mm^2^ with 95% CL of 684–801, which was slighter higher than that of microbead/hNP^IGF-TD^ group (724 × 10^4^ axons/mm^2^ and 95% CL of 670–775), but not statistically different (*P* > 0.05; Fig 6A and 6E and 6F). Both groups had similar anatomical distribution of axonal cross-sections. The axon densities of the two aforementioned groups were significantly higher than those of other groups receiving microbead/saline, microbead/hNP and microbead/hNP^TD^ (Fig 6B–6D and 6F). The values were 627, 648 and 643 axons/mm^2^, respectively with 95% CL of: 563–687, 579–710 and 587–695, respectively. Crosses in Fig 6F denote differences that reached a P value of 0.0512, SA group and 0.0902, TD group, whereas the NP group showed a P value of 0.1826 when all groups were compared with the NO group. Moreover, on optic cross-sections, many axons showed signs of degeneration including fiber disorganization and axon enlargement (Fig 6A–6E).

These observations confirm that IOP elevation without IGF-1 protection results in axon loss in optic nerve sections. Together, these results support the contention that IGF-1 supplied by a targeted cell delivery system can effectively preserve the RGC layer in the setting of experimental glaucoma. This effect seems to be specific to IGF-1 as neither hNP or hNP^TD^ cells can induce similar neuroprotection.

### Evaluation of Inflammatory and Angiogenic Pathways in Retinas after Microbead Injection

**Fig 7:**
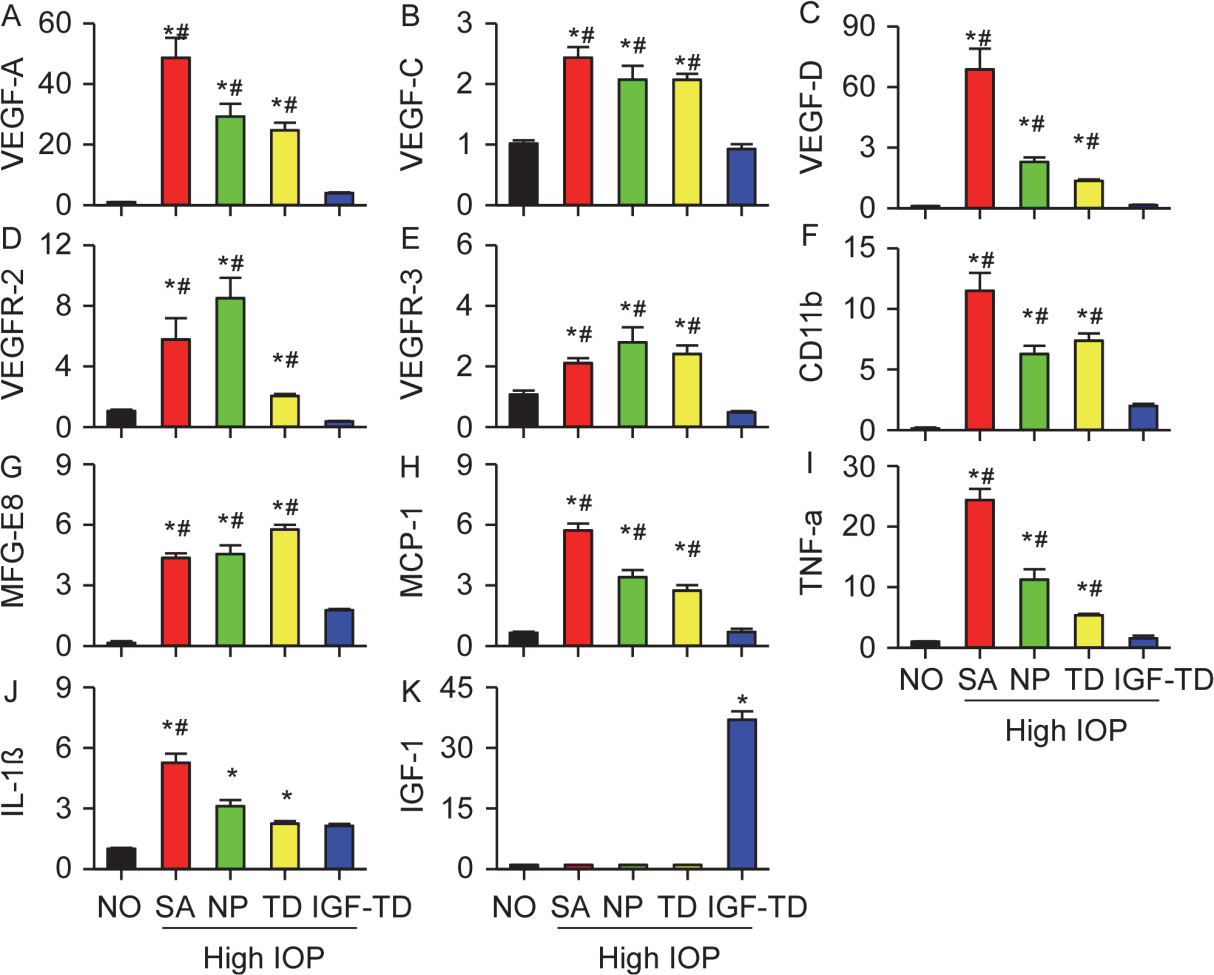
Quantification of angiogenic and inflammatory mRNA signals under various experimental conditions (data were presented as mean ± SD). Expression of genes related with angiogenic pathways (A—E), cell apoptosis (F—G) and inflammatory pathways (H—L) are shown. Significant increases in mRNA for typical genes related to angiogenic and inflammatory pathways after microbead injection is shown. These signals are tapered in microbead-injected eyes transplanted with hNP^IGF-TD^ cells. (K) IGF-1 component of the IGF-TD message can be detected in mice transplanted with hNP^IGF-TD^ cells (* *P* < 0.05 compared to NO group; # *P* < 0.05 compared to the mice transplanted with hNP^IGF-TD^ cells). Abbreviations: IGF-TD, transplanted hNP^IGF-TD^ cells after microbead injection; TD, transplanted hNP^TD^ cells after microbead injection. hNP, transplanted hNPs after microbead injection; SA, intravitreal saline (no cells) injection after microbead injection; NO, intravitreal saline injection and saline injection into the anterior chamber (no microbead and cell injection). High IOP, elevated intraocular pressure by microbead injection.

We studied the effects of hNP cell transplantation and secreted proteins, IGF-TD and TD, on gene expression in the host retina. We selected genes that were typically associated with apopototic, inflammatory and angiogenic pathways, listed in Table 1. In the microbead glaucoma groups (groups 2–4; saline, hNP and hNP^TD^), increased expression of the following genes was detected on day 30, as compared to baseline (all *P* < 0.05; Fig 7A–7J). These genes included VEGF-A, VEGF-D (increased 13-fold), VEGFR2, CD11b, MFG-E8 (macrophage receptor) and TNF-α.

These changes in gene expression were not observed in microbead glaucoma group that received hNP^IGF-TD^. In mice transplanted with hNP^IGF-TD^, expression of IGF-1 was expectedly higher than in other groups (Fig 7K, *P* < 0.05). In the 3 microbead glaucoma groups (saline, hNP and hNP^TD^) expression of CD11b and MFG-E8 microglial cell activation (which participate in RGC phagocytosis and apoptosis) were also upregulated (Fig 7F and 7G) and higher than their corresponding levels in the microbead glaucoma group receiving hNP^IGF-TD^ (*P* < 0.05). Interestingly, transplantation of hNP and hNP^TD^ exhibited some anti-inflammatory (TNF-α) and anti-angiogenic (VEGFs) effects. Overall gene expression experiments confirm that IGF-1 (in the form of IGF-TD) can significantly protect against RGC loss by affecting anti-inflammatory and anti- angiogenic pathways.

## Discussion

The current study shows proof-of-principle for targeted cell-based delivery of neurotropic factors, in this case, to the host RGC. In this scenario, hNPs that naturally hone into the inner retina layer, where the RGC cell bodies and nerve fibers lie, are programmed to secrete a factor of interest directly into the targeted sites. Our observations show that hNPs expressing IGF-TD penetrate the inner retinal layer and provide adequate neurotropic support to prevent glaucomatous RGC loss. Our proposed paradigm for cell-based therapy harbors intrinsic obstacles that need to be addressed and solved prior to initiating therapy, some of which have been addressed by this study. First, it is important to select a compatible cell line for targeted delivery. In our study, hNPs were a good choice because they spontaneously home to targeted tissue after intravitreal injection. Second, minimizing unwanted cell transformation. Cells require detailed characterized assuring that they do not transform and develop tumors. This has been an issue with induced pluripotent stem cells, which display high tendency to develop teratomas after transplantation [34,35]. We have extensively characterized hNPs and found that they have a very slow doubling time *in vitro* (approximately, every two weeks) and do not transform *in vitro* and *in vivo*. We were not able to develop teratomas or tumors after transplantation (unpublished observations). Third, candidate cells should be easy to transfect or infect and be able to express the factor of choice for an extended period of time. Localized low-level delivery of the factor is preferable to avoid side effects in distant tissues. It is known that IGF-1 could induce unwanted angiogenesis and retinal neovascularization [36]. This effect may be minimized by low-level targeted delivery.

For these experiments, we chose IGF-1 because it is a known and well-studied neurotropic factor. IGF-1 is primarily synthesized in the liver and plays an essential role in growth and development. IGF-1 is regarded as an important factor for regulation of cell growth and central nervous system development [37,38,39]. Studies have shown that IGF-1 and its receptor (IGF-1R) are able to stimulate growth in many different cell types and block apoptosis through providing proliferative signals [40]. *In vitro* studies have shown that IGF-1 can significantly enhance proliferation [41] and increase survival [42] of neurons, such as stimulating mitosis in sympathetic neuroblasts [43], mediating neuronal survival [44,45] and promoting neurite outgrowth of motor neurons [26]. The brain volume increases concomitantly with the increased expression of IGF-1 during postnatal development [19]. Similarly, there is a remarkable increase in neuronal numbers in IGF-1 transgenic mice. [36] Some studies indicate that the local density of axonal outgrowth within the brain is significantly increased in IGF-1 overexpressing mice [18,19]. In contrast, disruption of IGF-1 expression in homozygous knockout mice leads to brain growth retardation [19,46,47].

We evaluated the biological effects of IGF-1 on mouse primary RGCs in co-culture system, which allows us to investigate the function of IGF-1 on RGC survival and neurite outgrowth. We also evaluated the ability of hNPs expressing IGF-1 (fused to TD reporter protein) to confer trophic effects to host RGCs in the microbead model of mouse glaucoma. In support of this contention, previous studies have shown the utility of cells as carriers of specific genes and their products which can influence signaling pathways and gene expression [48,49]. However, these cells are delivered incased in carriers, or introduced either non-specifically into the therapeutic site or specifically using complicated surgical procedures [48,49]. hNPs have the intrinsic ability to hone into the therapeutic site within the inner retina, namely the RGC and nerve fiber layers after a simple intravitreal injection. This honing mechanism is chemotactic as pre-injection of the vitreous cavity with a glutamate toxin (*N*-methyl-*D*-asparte) abrogates penetration and integration of hNPs into the inner retina. In this scenario, hNPs continue to remain in the vitreous cavity (unpublished data).

In the glaucomatous eye, the expression of IGF-1 and other neurotropic factors in the neural retina is very limited as IGF-1 synthesis in the retina stops around time of birth and IGF-1 message is barely detectable thereafter. Nevertheless, there is some detectable retrograde transport of IGF-1 from the superior colliculus in the brain to RGC axons. This retrograde flow is severely diminished in glaucomatous eyes, suggesting that loss of retrograde IGF-1 may result in loss of trophic support and promote neurodegeneration [50]. Conversely, it is not surprising that supplementing IGF-1 through prolonged delivery to glaucomatous eyes may prevent at least some of the observed RGC degeneration. Our *in vitro* study demonstrates that cell-based delivery of IGF-1 dramatically increases the survival rates and neurite outgrowth of primary RGCs, and that hNPs could be used as an efficient delivery vehicle to continuously provide IGF-1 support.

These observations formed the basis of our study in which we employed hNPs transfected with vectors carrying IGF-TD to deliver biologically active IGF-1 fusion protein. Also, the microbead model of rodent glaucoma is indeed highly reproducible resulting in loss of RGCs and thinning of the nerve fiber layer. Our findings are in line with previous studies [29,30]. The degree of RGC loss was similar among the mouse groups that received no cells, hNPs or hNP^TD^, confirming that the active agent was indeed IGF-1 and hNPs do not secrete appropriate or adequate levels of their own cytokines to prevent axon loss. We cannot rule out any anti-angiogenic effects attributed to hNPs as our gene expression studies do show lower levels of VEGFs in hNP and hNP^TD^ transplanted eyes. Perhaps, the combination of hNP with the IGF-TD expression vector is the key ingredient to suppress RGC loss in experimental glaucoma.

Based on our observations in the RT-PCR experiments that showed upregulation of inflammatory message in glaucoma groups which was inhibited by IGF-TD, we propose a role for upregulation of inflammatory pathways in the pathogenesis of RGC loss in the setting of glaucoma. In the saline injected glaucoma group (group 2), over 5 to 20 fold increase in the messages for TNF-α, IL-1ß and MCP-1 were detected as compared with the non-glaucomatous saline group. In contrast, in the hNP^IGF-TD^ group the levels of TNF-α, IL-1ß and MCP-1 were slightly higher but not statistically different from non-glaucomatous saline group. Interestingly, the levels of these cytokines were tapered in the hNP and hNP^TD^ groups suggesting that transplantation of cells themselves exert some intrinsic anti-inflammatory effect as well.

RT-PCR data also show tapering of the angiogenic pathways. Several studies have shown that IGF-1 could induce neovascularization and other related pathology [31,32]. Native IGF-1 is considered a mitogen that may stimulate [32,51,52] or support [53] the development of retinal neovascularization, possibly through upregulation of vascular endothelial growth factor. In the saline-injected glaucoma group (group 2) increased expression of VEGF-A, C and D and cognate receptors, VEGFR-2 and R-3 were detected, as compared with the no glaucoma group (group 1). In the hNP^IGF-TD^ group, the levels of these cytokines and receptors were tapered and closer to the no glaucoma (group 1). In the glaucoma groups transplanted with hNP and hNP^TD^, the levels of VEGF-C, VEGFR-2 and R-3 were similar to saline-injected glaucoma group (group 2). Interestingly, the levels of VEGF-A and D were tapered suggesting that hNP cells have an intrinsic ability to down-regulate these pathways.

Our data are in contrast to prior studies that have implicated IGF-1 as a pro-angiogenic factor.^39^ Our observations suggest that activation of IGF-1R (through IGF-TD) negatively regulates inflammatory and angiogenic pathways; these may independent of neurotrophic pathways stimulated by IGF-TD. This is in concert with previous studies that have indicated that neovascularization is almost always accompanied by inflammation [54,55]. Angiogenic and inflammatory pathways may go hand in hand in the glaucomatous eye regardless of whether xenografts (human cells) are introduced. It is likely that the microenvironment of the glaucomatous eye promotes a pro-inflammatory and pro-angiogenic environment, which is negatively regulated by IGF-1 signaling pathways and acts concurrently to activation of NTFs.

As an effort to dissect IGF-1-regulated pathways, previous studies have shown that one of these pathways may involve phosphoinositide 3-kinase (PI3K) signaling [56,57]. IGF-1 through activation of its receptor, IGF-R1 promotes survival of neurons via the PI3K pathway [58,59,60]. Our *in vitro* study indicated that the survival rate of primary cultured RGCs significantly decreased when inhibitors of IGF-1R signaling pathways were applied. These findings are in accord with previous studies that demonstrate that blockage of IGF-1R signaling interrupts survival [39,43,61,62].

We observed that microbead-injected glaucomatous eyes developed a pro-inflammatory milieu (even in absence of cell transplantation; unpublished observations). We have therefore hypothesized that elevation of IOP may setup inflammatory responses which may lead up to RGC death. Downregulation of inflammatory pathways by IGF-1 may be one mechanism for its neurotropic effects. In corollary to this, we have observed increased inflammatory cells on histological eyes from DBA/2J mice that develop very elevated IOPs [63,64]. TNF-α may be a strong participant in this process. Recent evidence suggests that TNF-α promotes neurodegeneration through inhibition of IGF-1 pathways [65,66]. By inhibiting essential components of IGF-1 downstream regulators, such as PI3K, low non-toxic concentrations of TNF-α secreted by reactivated glial cells may indirectly trigger the death of neurons [67,68,69]. Some studies have shown increased levels of TNF-α in glaucomatous retinas obtained at autopsy [70]. Tezel et al. found that up-regulation of TNF-α and its receptor-1 in glaucomatous retina suggesting that TNF-α-mediated cell death is involved in the neurodegeneration processes in glaucoma [70]. Increased production of TNF-α by glial cells in glaucomatous eyes may therefore lead to death of RGCs through direct activation of the apoptotic cell death cascade [70]. Tezel and Wax found that glial cell secrete TNF-α as well as other noxious agents such as nitric oxide into co-culture media, which in turn facilitate apoptotic death of RGCs [69]. Their findings suggest that inhibition of TNF-α secreted by reactivated glial cells may provide a new therapeutic target for neuroprotection in the treatment of glaucomatous optic neuropathy [69].

IGF-1 can also reduce the production of pro-inflammatory cytokines through suppression of NF-κB signaling pathway, thus inhibiting the cascade of pro-inflammatory cytokines [71]. Our results indicate that hNP^IGF-TD^ cells reduce production of other chemokines, such as MCP-1, suggesting that hNP^IGF-TD^ cells can influence chemokine production and decrease recruitment of peripheral leukocytes [72]. The levels of messages for pro-inflammatory cytokines, TNF-α and IL-1ß were quite low in the glaucomatous mice transplanted with hNP^IGF-TD^ cells.

An interesting observation involves the effects of hNPs (including hNP^TD^) in absence of any IGF-1 on experimental glaucoma. Although these eyes exhibited loss of RGCs and axonal damage similar to those observed in the saline injected microbead group, hNPs did influence the message for inflammatory factors but had a modest anti-angiogenic effect by affecting VEGF levels. It is likely that the combination of IGF-1 and hNPs may be important for maintaining an anti-inflammatory and anti-angiogenic milieu in the setting of glaucoma.

In summary, this study describes the application of a specialized neuronal progenitor cells line that spontaneously hones in to the inner retinal layers as a vehicle for local production and delivery of a desired NTF. The aim of neuroprotection for glaucoma therapy is to use agents that prevent or delay RGC death, as well as rescue or even support regeneration of already compromised RGCs. Delivery of NTFs does not have be executed in a non-specific, gunshot manner. Low-dose targeted delivery of NTFs such as IGF-1 released transplanted cells is adequate to confer meaningful neuroprotection. We have shown that hNPs are good cell candidates for RGC rescue as they can spontaneously penetrate and integrate into the host RGC and nerve fiber layers where they locally synthesize and secret biologically active IGF-1 [11]. Our findings from the co-culture systems also show that IGF-1 has significant positive effects on enhancing RGC survival rate and neurite growth. Transplantation of hNP^IGF-TD^ cells effectively protects survival of host RGCs after microbead injection. These findings have provided experimental evidence and form the basis for applying cell-based strategies for local delivery of NTFs into the retina. These applications may be extended to other disease conditions beyond glaucoma.

## References

[1] MacLaren RE, Pearson RA, MacNeil A, Douglas RH, Salt TE, Akimoto M, et al (2006) Retinal repair by transplantation of photoreceptor precursors. Nature 444: 203–207. 17093405 10.1038/nature05161

[2] Pearson RA, Barber AC, Rizzi M, Hippert C, Xue T, West EL, et al (2012) Restoration of vision after transplantation of photoreceptors. Nature 485: 99–103. 10.1038/nature10997 22522934 PMC3888831

[3] Pearson RA (2014) Advances in repairing the degenerate retina by rod photoreceptor transplantation. Biotechnology Advances 32: 485–491. 10.1016/j.biotechadv.2014.01.001 24412415 PMC4070022

[4] Barber AC, Hippert C, Duran Y, West EL, Bainbridge JWB, Warre-Cornish K, et al (2013) Repair of the degenerate retina by photoreceptor transplantation. Proceedings of the National Academy of Sciences of the United States of America 110: 354–359. 10.1073/pnas.1212677110 23248312 PMC3538261

[5] Mead B, Logan A, Berry M, Leadbeater W, Scheven BA (2014) Paracrine-mediated neuroprotection and neuritogenesis of axotomised retinal ganglion cells by human dental pulp stem cells: comparison with human bone marrow and adipose-derived mesenchymal stem cells. PLoS ONE 9: e109305 10.1371/journal.pone.0109305 25290916 PMC4188599

[6] Mead B, Logan A, Berry M, Leadbeater W, Scheven BA (2013) Intravitreally transplanted dental pulp stem cells promote neuroprotection and axon Regeneration of retinal ganglion cells after optic nerve injury. Investigative Ophthalmology & Visual Science 54: 7544–7556. 24150755 10.1167/iovs.13-13045

[7] Johnson TV, Bull ND, Hunt DP, Marina N, Tomarev SI, Martin KR (2010) Neuroprotective effects of intravitreal mesenchymal stem cell transplantation in experimental glaucoma. Investigative Ophthalmology & Visual Science 51: 2051–2059. 19933193 10.1167/iovs.09-4509 PMC2868400

[8] Suzuki K, Murtuza B, Beauchamp JR, Brand NJ, Barton PJR, Varela-Carver A, et al (2004) Role of interleukin-1β in acute inflammation and graft death after cell transplantation to the heart. Circulation 110: II-219–II-224. 15364866 10.1161/01.CIR.0000138388.55416.06

[9] Goldman D (2014) Muller glial cell reprogramming and retina regeneration. Nat Rev Neurosci 15: 431–442. 10.1038/nrn3723 24894585 PMC4249724

[10] Lenkowski JR, Raymond PA (2014) Müller glia: Stem cells for generation and regeneration of retinal neurons in teleost fish. Progress in Retinal and Eye Research 40: 94–123. 10.1016/j.preteyeres.2013.12.007 24412518 PMC3999222

[11] Chen GC, Ma J, Shatos MA, Chen HH, Cyr DE, Lashkari K (2012) Application of human persistent fetal vasculature neural progenitors for transplantation in the inner retina. Cell Transplantation 21: 2621–2634. 10.3727/096368912X647153 23317920

[12] Kermer P, Klöcker N, Labes M, Bähr M (2000) Insulin-like growth factor-I protects axotomized rat retinal ganglion cells from secondary death via PI3-K-dependent Akt phosphorylation and inhibition of caspase-3 *in vivo* . The Journal of Neuroscience 20: 722–728. 10.1523/JNEUROSCI.20-02-00722.2000 PMC6772394 10632601

[13] Liu XJ, Xie Q, Zhu YF, Chen C, Ling N (2001) Identification of a nonpeptide ligand that releases bioactive insulin-like growth factor-I from Its binding protein complex. The Journal of Biological Chemistry 276: 32419–32422. 11445558 10.1074/jbc.C100299200

[14] Ren J, Duan J, Hintz KK, Ren BH (2003) High glucose induces cardiac insulin-like growth factor I resistance in ventricular myocytes: role of Akt and ERK activation. Cardiovascular Research 57: 738–748. 12618235 10.1016/s0008-6363(02)00788-5

[15] Burren CP, Berka JL, Edmondson SR, Werther GA, Batch JA (1996) Localization of mRNAs for insulin-like growth factor-I (IGF-I), IGF-I receptor, and IGF binding proteins in rat eye. Investigative Ophthalmology & Visual Science 37: 1459–1468. 8641849

[16] Danias J, Stylianopoulou F (1996) Expression of IGF-I and IGF-II genes in the adult rat eye. Current Eye Research 9: 379–386. 10.3109/02713689008999626 1692782

[17] Batchelor DC, Hutchins A-M, Klempt M, Skinner SJM (1995) Developmental changes in the expression patterns of IGFs, type 1 IGF receptor and IGF-binding proteins-2 and -4 in perinatal rat lung. Journal of Molecular Endocrinology 15: 105–115. 8800636 10.1677/jme.0.0150105

[18] Ye P, Carson J, D'Ercole A (1995) In vivo actions of insulin-like growth factor-I (IGF-I) on brain myelination: studies of IGF-I and IGF binding protein-1 (IGFBP-1) transgenic mice. The Journal of Neuroscience 15: 7344–7356. 7472488 10.1523/JNEUROSCI.15-11-07344.1995 PMC6578047

[19] O'Kusky JR, Ye P, D'Ercole AJ (2000) Insulin-like growth factor-I promotes neurogenesis and synaptogenesis in the hippocampal dentate gyrus during postnatal development. The Journal of Neuroscience 20: 8435–8442. 11069951 10.1523/JNEUROSCI.20-22-08435.2000 PMC6773150

[20] Laron Z (1999) Somatomedin-1 (recombinant Insulin-like growth factor-1): clinical pharmacology and potential treatment of endocrine and metabolic disorders. BioDrugs 11: 55–70. 18031115 10.2165/00063030-199911010-00006

[21] Guler H-P, Zapf J, Schmid C, Froesch ER (1989) Insulin-like growth factors I and II in healthy man. Acta Endocrinologica 121: 753–758. 2558477 10.1530/acta.0.1210753

[22] Knappskog S, Ravneberg H, Gjerdrum C, Tröβe C, Stern B, Pryme IF (2007) The level of synthesis and secretion of Gaussia princeps luciferase in transfected CHO cells is heavily dependent on the choice of signal peptide. Journal of Biotechnology 128: 705–715. 17316861 10.1016/j.jbiotec.2006.11.026

[23] Ruecker O, Zillner K, Groebner-Ferreira R, Heitzer M (2008) Gaussia-luciferase as a sensitive reporter gene for monitoring promoter activity in the nucleus of the green alga *Chlamydomonas reinhardtii* . Molecular Genetics and Genomics 280: 153–162. 10.1007/s00438-008-0352-3 18516621

[24] Engvall E, Perlmann P (1971) Enzyme-linked immunosorbent assay (ELISA) quantitative assay of immunoglobulin G. Immunochemistry 8: 871–874. 5135623 10.1016/0019-2791(71)90454-x

[25] Douglas AD, Williams AR, Illingworth JJ, Kamuyu G, Biswas S, Goodman AL, et al (2011) The blood-stage malaria antigen PfRH5 is susceptible to vaccine-inducible cross-strain neutralizing antibody. Nature Communications 2: 601 10.1038/ncomms1615 22186897 PMC3504505

[26] Özdinler PH, Macklis JD (2006) IGF-I specifically enhances axon outgrowth of corticospinal motor neurons. Nature Neuroscience 9: 1371–1381. 17057708 10.1038/nn1789

[27] Pietrzkowski Z, Wernicke D, Porcu P, Jameson BA, Baserga R (1992) Inhibition of cellular proliferation by peptide analogues of insulin-like growth factor 1. Cancer Research 52: 6447–6451. 1423292

[28] Hintz KK, Ren J (2002) Prediabetic insulin resistance is not permissive to the development of cardiac resistance to insulin-like growth factor I in ventricular myocytes. Diabetes Research and Clinical Practice 55: 89–98. 11796174 10.1016/s0168-8227(01)00323-0

[29] Chen HH, Wei X, Cho KS, Chen GC, Sappington R, Calkins DJ, et al (2011) Optic neuropathy due to microbead-induced elevated intraocular pressure in the mouse. Investigative Ophthalmology & Visual Science 52: 36–44. 20702815 10.1167/iovs.09-5115 PMC3053285

[30] Yang Q, Cho KS, Chen HH, Yu D, Wang WH, Luo G, et al (2012) Microbead-induced ocular hypertensive mouse model for screening and testing of aqueous production suppressants for glaucoma. Investigative Ophthalmology & Visual Science 53: 3733–3741. 22599582 10.1167/iovs.12-9814 PMC3390181

[31] Kondo T, Vicent D, Suzuma K, Yanagisawa M, King GL, Holzenberger M, et al (2003) Knockout of insulin and IGF-1 receptors on vascular endothelial cells protects against retinal neovascularization. The Journal of Clinical Investigation 111: 1835–1842. 12813019 10.1172/JCI17455 PMC161423

[32] Chen J, Smith LH (2007) Retinopathy of prematurity. Angiogenesis 10: 133–140. 17332988 10.1007/s10456-007-9066-0

[33] Liu Y, Yang X, Utheim TP, Guo C, Xiao M, Liu Y, et al (2013) Correlation of cytokine levels and microglial cell infiltration during retinal degeneration in RCS rats. PLoS ONE 8: e82061 10.1371/journal.pone.0082061 24349184 PMC3862575

[34] Ben-David U, Benvenisty N (2011) The tumorigenicity of human embryonic and induced pluripotent stem cells. Nature Reviews Cancer 11: 268–277. 10.1038/nrc3034 21390058

[35] Gutierrez-Aranda I, Ramos-Mejia V, Bueno C, Munoz-Lopez M, Real PJ, Mácia A, et al (2010) Human induced pluripotent stem cells develop teratoma more efficiently and faster than human embryonic stem cells regardless the site of injection. Stem Cells 28: 1568–1570. 10.1002/stem.471 20641038 PMC2996086

[36] Behringer RR, Lewin TM, Quaife CJ, Palmiter RD, Brinster RL, D’Ercole AJ (1990) Expression of insulin-like growth factor I stimulates normal somatic growth in growth hormone-deficient transgenic mice. Endocrinology 127: 1033–1040. 2387246 10.1210/endo-127-3-1033

[37] Wilkins A, Chandran S, Compston A (2001) A role for oligodendrocyte-derived IGF-1 in trophic support of cortical neurons. Glia 36: 48–57. 11571783 10.1002/glia.1094

[38] Ye P, Umayahara Y, Ritter D, Bunting T, Auman H, Rotwein P, et al (1997) Regulation of insulin-like growth factor I (IGF-I) gene expression in brain of transgenic mice expressing an IGF-I-luciferase fusion gene. Endocrinology 138: 5466–5475. 9389533 10.1210/endo.138.12.5600

[39] Popken GJ, Hodge RD, Ye P, Zhang J, Ng W, O'Kusky JR, et al (2004) In vivo effects of insulin-like growth factor-I (IGF-I) on prenatal and early postnatal development of the central nervous system. European Journal of Neuroscience 19: 2056–2068. 15090033 10.1111/j.0953-816X.2004.03320.x

[40] Galvan V, Logvinova A, Sperandio S, Ichijo H, Bredesen DE (2003) Type 1 insulin-like growth factor receptor (IGF-IR) signaling inhibits apoptosis signal-regulating kinase 1 (ASK1). The Journal of Biological Chemistry 278: 13325–13332. 12556535 10.1074/jbc.M211398200

[41] Bondy CA, Lee W-H (1993) Patterns of insulin-like growth factor and IGF receptor gene expression in the brain: functional implications. Annals of the New York Academy of Sciences 692: 33–43. 8215043 10.1111/j.1749-6632.1993.tb26203.x

[42] Dobrowolny G, Giacinti C, Pelosi L, Nicoletti C, Winn N, Barberi L, et al (2005) Muscle expression of a local Igf-1 isoform protects motor neurons in an ALS mouse model. The Journal of Cell Biology 168: 193–199. 15657392 10.1083/jcb.200407021 PMC2171577

[43] DiCicco-Bloom E, Black IB (1988) Insulin growth factors regulate the mitotic cycle in cultured rat sympathetic neuroblasts. Proceedings of the National Academy of Sciences of the United States of America 85: 4066–4070. 2897692 10.1073/pnas.85.11.4066 PMC280362

[44] Fadok VA, Bratton DL, Rose DM, Pearson A, Ezekewitz RAB, Henson PM (2000) A receptor for phosphatidylserine-specific clearance of apoptotic cells. Nature 405: 85–90. 10811223 10.1038/35011084

[45] Hanayama R, Tanaka M, Miwa K, Shinohara A, Iwamatsu A, Nagata S (2002) Identification of a factor that links apoptotic cells to phagocytes. Nature 417: 182–187. 12000961 10.1038/417182a

[46] Baker J, Liu J-P, Robertson EJ, Efstratiadis A (1993) Role of insulin-like growth factors in embryonic and postnatal growth. Cell 75: 73–82. 8402902

[47] Liu J, Baker J, Perkins AS, Robertson EJ, Efstratiadis A (1993) Mice carrying null mutations of the genes encoding insulin-like growth factor I (Igf-1) and type 1 IGF receptor (Igf1r). Cell 75: 59–72. 8402901

[48] Xu LC, Karlsson S, Byrne ER, Kluepfel-Stahl S, Kessler SW, Agricola BA, et al (1995) Long-term in vivo expression of the human glucocerebrosidase gene in nonhuman primates after CD34+ hematopoietic cell transduction with cell-free retroviral vector preparations. Proceedings of the National Academy of Sciences of the United States of America 92: 4372–4376. 7538667 10.1073/pnas.92.10.4372 PMC41946

[49] Bienzle D, Abrams-Ogg AC, Kruth SA, Ackland-Snow J, Carter RF, Dick JE, et al (1994) Gene transfer into hematopoietic stem cells: long-term maintenance of in vitro activated progenitors without marrow ablation. Proceedings of the National Academy of Sciences of the United States of America 91: 350–354. 8278392 10.1073/pnas.91.1.350 PMC42945

[50] Guo L, Moss SE, Alexander RA, Ali RR, Fitzke FW, Cordeiro MF (2005) Retinal ganglion cell apoptosis in glaucoma is related to intraocular pressure and IOP-induced effects on extracellular matrix. Investigative Ophthalmology & Visual Science 46: 175–182. 15623771 10.1167/iovs.04-0832 PMC2601028

[51] Meyer-Schwickerath R, Pfeiffer A, Blum WF, Freyberger H, Klein M, Lösche C, et al (1993) Vitreous levels of the insulin-like growth factors I and II, and the insulin-like growth factor binding proteins 2 and 3, increase in neovascular eye disease. Studies in nondiabetic and diabetic subjects. The Journal of Clinical Investigation 92: 2620–2625. 7504689 10.1172/JCI116877 PMC288458

[52] Grant M, Russell B, Fitzgerald C, Merimee T (1986) Insulin-like growth factors in vitreous. Studies in control and diabetic subjects with neovascularization. Diabetes 35: 16–20. 10.2337/diab.35.4.416 2420665

[53] Smith L, Kopchick J, Chen W, Knapp J, Kinose F, Daley D, et al (1997) Essential role of growth hormone in ischemia-induced retinal neovascularization. Science 276: 1706–1709. 9180082 10.1126/science.276.5319.1706

[54] Epstein RJ, Stulting RD, Hendricks RL, Harris DM (1987) Corneal neovascularization. Pathogenesis and inhibition. Cornea 6: 250–257. 2446823 10.1097/00003226-198706040-00004

[55] Dana MR (2006) Angiogenesis and lymphangiogenesis—Implications for corneal Immunity. Seminars in Ophthalmology 21: 19–22. 16517440 10.1080/08820530500509358

[56] Heck S, Lezoualc’h F, Engert S, Behl C (1999) Insulin-like growth factor-1-mediated neuroprotection against oxidative stress is associated with activation of nuclear factor κB. The Journal of Biological Chemistry 274: 9828–9835. 10092673 10.1074/jbc.274.14.9828

[57] Seigel GM, Chiu L, Paxhia A (2000) Inhibition of neuroretinal cell death by insulin-like growth factor-1 and its analogs. Mol Vis 6: 157–163. 10973501

[58] Dudek H, Datta SR, Franke TF, Birnbaum MJ, Yao R, Cooper GM, et al (1997) Regulation of neuronal Survival by the serine-threonine protein kinase Akt. Science 275: 661–665. 9005851 10.1126/science.275.5300.661

[59] Dupraz S, Grassi D, Karnas D, Nieto Guil AF, Hicks D, Quiroga S (2013) The Insulin-like growth factor 1 receptor is essential for axonal regeneration in adult central nervous system neurons. PLoS ONE 8: e54462 10.1371/journal.pone.0054462 23349896 PMC3548777

[60] O'Driscoll C, Donovan M, Cotter TG (2006) Analysis of apoptotic and survival mediators in the early post-natal and mature retina. Experimental Eye Research 83: 1482–1492. 17011550 10.1016/j.exer.2006.08.007

[61] Kwon J, Stephan S, Mukhopadhyay A, Muders MH, Dutta SK, Lau JS, et al (2009) Insulin receptor substrate-2 mediated insulin-like growth factor-I receptor overexpression in pancreatic adenocarcinoma through protein kinase Cδ. Cancer Research 69: 1350–1357. 10.1158/0008-5472.CAN-08-1328 19190347 PMC2705142

[62] White MF, Shoelson SE, Keutmann H, Kahn CR (1988) A cascade of tyrosine autophosphorylation in the beta-subunit activates the phosphotransferase of the insulin receptor. The Journal of Biological Chemistry 263: 2969–2980. 2449432

[63] Zhou X, Li F, Kong L, Tomita H, Li C, Cao W (2005) Involvement of inflammation, degradation, and apoptosis in a mouse model of glaucoma. Journal of Biological Chemistry 280: 31240–31248. 15985430 10.1074/jbc.M502641200

[64] Bosco A, Inman DM, Steele MR, Wu G, Soto I, Marsh-Armstrong N, et al (2008) Reduced retina microglial activation and improved optic nerve integrity with minocycline treatment in the DBA/2J mouse model of glaucoma. Investigative Ophthalmology & Visual Science 49: 1437–1446. 18385061 10.1167/iovs.07-1337

[65] Peraldi P, Hotamisligil GS, Buurman WA, White MF, Spiegelman BM (1996) Tumor necrosis factor (TNF)-α inhibits insulin signaling through stimulation of the p55 TNF receptor and activation of sphingomyelinase. Journal of Biological Chemistry 271: 13018–13022. 8662983 10.1074/jbc.271.22.13018

[66] Paz K, Hemi R, LeRoith D, Karasik A, Elhanany E, Kanety H, et al (1997) A molecular basis for insulin resistance: elevated serine/threonine phosphorylation of IRS-1 and IRS-2 inhibits their binding to the juxtamembrane region of the insulin receptor and impairs their ability to undergo insulin-induced tyrosine phosphorylation. Journal of Biological Chemistry 272: 29911–29918. 9368067 10.1074/jbc.272.47.29911

[67] Venters HD, Dantzer R, Kelley KW (2000) Tumor necrosis factor-α induces neuronal death by silencing survival signals generated by the type I insulin-like growth factor receptor. Annals of the New York Academy of Sciences 917: 210–220. 11268346 10.1111/j.1749-6632.2000.tb05385.x

[68] Boyd ZS, Kriatchko A, Yang J, Agarwal N, Wax MB, Patil RV (2003) Interleukin-10 receptor signaling through STAT-3 regulates the apoptosis of retinal ganglion cells in response to stress. Investigative Ophthalmology & Visual Science 44: 5206–5211. 14638718 10.1167/iovs.03-0534

[69] Tezel G, Wax MB (2000) Increased production of tumor necrosis factor-α by glial cells exposed to simulated ischemia or elevated hydrostatic pressure induces apoptosis in cocultured retinal ganglion cells. The Journal of Neuroscience 20: 8693–8700. 11102475 10.1523/JNEUROSCI.20-23-08693.2000 PMC6773089

[70] Tezel G, Li LY, Patil RV, Wax MB (2001) TNF-α and TNF-α receptor-1 in the retina of normal and glaucomatous eyes. Investigative Ophthalmology & Visual Science 42: 1787–1794. 11431443

[71] Choi H, Lee RH, Bazhanov N, Oh JY, Prockop DJ (2011) Anti-inflammatory protein TSG-6 secreted by activated MSCs attenuates zymosan-induced mouse peritonitis by decreasing TLR2/NF-κB signaling in resident macrophages. Blood 118: 330–338. 10.1182/blood-2010-12-327353 21551236 PMC3138686

[72] Zhang R, Liu Y, Yan K, Chen L, Chen XR, Li P, et al (2013) Anti-inflammatory and immunomodulatory mechanisms of mesenchymal stem cell transplantation in experimental traumatic brain injury. Journal of Neuroinflammation 10: 106 10.1186/1742-2094-10-106 23971414 PMC3765323

